# Factors in the Ætiology of Disseminated Sclerosis
*A paper read to the Bristol Medico-Chirurgical Society on 14th January, 1948.


**Published:** 1948

**Authors:** A. M. G. Campbell

**Affiliations:** Physician, United Bristol Hospitals


### FACTORS IN THE ÆTIOLOGY OF DISSEMINATED SCLEROSIS*

BY

A. M. G. CAMPBELL, D.M., M.R.C.P.

*Physician, United Bristol Hospitals.*

---

THE purpose of this communication is to indicate recent lines of thought about Disseminated Sclerosis. The ætiology of this disease has remained a complete mystery. It has been suggested in the past, by Bing[1] in Switzerland and Säalström[2] in Sweden, that this disease had a geographical incidence. Out of the general impressions of these and other writers, it has appeared that it is much more common in the northern than in the southern hemisphere. For example, it is said to occur more commonly in the United States than in South America. Furthermore, reference to South African neurologists has emphasized its extreme rarity in native-born South Africans ; I have personally heard from most of the leading South African neurologists that this is, in fact, the case, although cases from Europe may develop a relapse in South Africa.

Recently, Snapper[3] has stated that the disease is very rare in China, amongst both Chinese and Europeans. It has been said with some truth that the incidence of Disseminated Sclerosis is directly proportional to the incidence of neurologists in the area concerned, but this hardly explains the concurrence of opinion from South Africa and the views of so careful an observer as Snapper.

These views led us to try to estimate the geographical incidence in this country. A small survey had been made by Allison[4] in 1931, and we attempted a bigger survey on the basis of a questionnaire sent to doctors in Oxfordshire, Buckinghamshire, Berkshire, Cornwall, Suffolk and Derbyshire. We asked the doctors to notify us of their definite cases only, and cases which had been resident in their area for at least two years. The inaccuracies of such a survey are enormous, as it is impossible to visit all the cases, but in our team at Oxford we personally visited a large number of cases, and as a matter of interest found the diagnoses to be correct in the great majority of cases. We felt that this was because the chronicity of the disease ultimately led to a diagnosis by a specialist.

One of the questions asked was the possible relationship in onset

* A paper read to the Bristol Medico-Chirurgical Society on 14th January, 1948.

to pregnancy, and about 10 per cent. of the case returns showed this to be a factor either in the onset or the relapse. As regards the geographical incidence, the figures for incidence were remarkably constant, with one exception. We divided the country into population areas of roughly 60,000, and on the whole the incidence was remarkably constant at two cases for 10,000 of the population. In the Bakewell district of Derbyshire, to which I shall refer later as the area where Swayback disease of lambs is commonest, the incidence was more like five to six cases per 10,000 persons. In no area was the disease absent.

This figure of two per 10,000 is approximately the same incidence as in Sweden, Switzerland and the United States of America. In our survey, however, a more interesting fact was the occurrence of groups of cases in small villages, where, compared to a rate of two per 10,000, the incidences were extremely high. It is to these groups of cases that our attention is now directed in an effort to explain this high incidence.

It is too early to draw conclusions, but if any value is to come from a geographical survey it must be from a study of small communities. We feel a large postal questionnaire is valueless except to indicate a high incidence in small areas for further study. People move about so much that any study not undertaken on a more or less stationary community may be of little value. We once visited six cases in one road and found none had lived more than five years in that vicinity. The studies in Sweden and Switzerland have all suffered from too general an approach.

Our investigations led up one other path which may yield some help. There occurs in many parts of the world a demyelinating disease of lambs called in this country, " Swayback ". It has a strictly geographical incidence and may occur on one farm whilst five miles away the flocks are free from it. In England, as I mentioned previously, it is most commonly found in the Bakewell district of Derbyshire, and here there are certain farms where it has been impossible to breed sheep because of the high mortality in lambs. An unaffected flock brought to this area will, in eighteen months, throw affected lambs. It must be emphasized that the ewes are clinically unaffected, and it is only their offspring that show, about the third week of life, the characteristic motor weakness and ataxia. Pathologically the disease resembles Schilder's disease in children, in that large cavities appear in the white matter of the brain. It does not, therefore, except in being a demyelinating condition, resemble Disseminated Sclerosis very closely.

A remarkable discovery was made in 1936 by Bennetts[5] in Australia, who found that this disease was associated with a low copper content in both the ewe and the lamb, and that in Australia this was due to a deficiency in copper in the herbage. Innes[6] and his co-workers working on Derbyshire flocks confirmed the low copper findings in the animals, but failed to find low copper in the herbage, and suggested a failure to utilize copper. They also found in the Derbyshire cases a high lead content of the brains and liver in lambs and ewes affected. Finally, by feeding copper sulphate in the form of licks to affected lambs, the disease could be completely controlled. Here, therefore, is the only demyelinating disease which we can at least prevent.

This led us to consider this disease from the point of view of Disseminated Sclerosis. Both Swayback and Disseminated Sclerosis have a possible geographical incidence and we also have the high incidence of Disseminated Sclerosis in the Bakewell area of Derbyshire, but the relationship appeared somewhat slender until we discovered one most extraordinary fact, which appeared more than coincidental. Four of the six veterinary pathologists working at Cambridge on the Swayback problem from 1934 to 1938 developed clinical signs typical of Disseminated Sclerosis. I have sketched the history of these men in a previous paper[7] and it suffices to say that all showed dissemination of symptoms, and relapses and remissions of the disease. Whether the disease is true Disseminated Sclerosis one cannot say, but all the cases had been independently diagnosed as such by other neurologists, before we saw them. An explanation of this extraordinary association is extremely difficult. All these men were connected with post-mortem material and also had worked in the vicinity of Swayback farms. It is possible that they were affected by some agent from the post-mortem material or at least were subjected to exposure to a toxic agent during this work, either in the Swayback area or the laboratory. We have been unable to explain this high incidence of a somewhat rare disease amongst the workers.

It is therefore along these lines that we have been working, that is, a geographical incidence, and the possible implication of trace elements. It can be seen that lead as a causative factor is not entirely ruled out and lead may have some bearing on copper metabolism. The next step in the elucidation of Disseminated Sclerosis must lie in the understanding of the chemical and pathological processes underlying demyelination, for until we understand this the subject will remain complex.

In conclusion it may be said that Russian workers[8] have recently claimed to have discovered a virus in Disseminated Sclerosis, but they have refused to allow any of it to be tested by workers outside "The Iron Curtain", so that we remain uncertain of their claims. Disseminated Sclerosis is not a very rare disease, it appears common in the South-Western area and it still remains unsolved: perhaps by the united efforts of the general practitioner, research worker, veterinary pathologist and soil scientist, the problem may become clearer. It is my view that lead may play some part in the ætiology of Disseminated Sclerosis and Swayback and that the seeds of Disseminated Sclerosis may be sown years before the development of the clinical symptoms. If this be correct, a careful history on each case of Disseminated Sclerosis is essential.

### REFERENCES

[1] Bing, R. Die Multiple Sklerose einst und jetzt. Basel, 1932.

[2] Säalström, T. (1942), *Acta. Med. Scandinav.* supp. 137, p.1.

[3] Snapper, T (1946), Personal Communication.

[4] Allison, R. S. (1931), *Brain*, 54, 281.

[5] Bennetts, H. W. (1937), *Austral. Vet. Journ.*, 18, 50.

[6] Innes, J. R. M. (1940), *Journ. Comp. Path.*, 53, 1.

[7] Campbell, A. M. G., Porter, R. W., Russell, R. W. (1947) *Brain*, 71, 18.

[8] Margulis, Soloviev and Shubladze (1946), *Jour. Neurol. Neurosurg. and Psychiat.*, 9, 63.

